# Exploring the link between cadmium and psoriasis in a nationally representative sample

**DOI:** 10.1038/s41598-017-01827-9

**Published:** 2017-05-11

**Authors:** Fang-Yih Liaw, Wei-Liang Chen, Tung-Wei Kao, Yaw-Wen Chang, Ching-Fu Huang

**Affiliations:** 1 Family Medicine and Geriatric Medicine, Department of Family and Community health, Tri-Service General Hospital, National Defense Medical Center, Taipei, Taiwan; 2 Graduate Institute of Medical Sciences, National Defense Medical Center, Taipei, Taiwan; 3 Graduate Institute of Clinical Medicine, College of Medicine, National Taiwan University, Taipei, Taiwan; 4 Department of Dermatology, Taoyuan Armed Forces General Hospital, Taoyuan and National Defense Medical Center, Taipei, Taiwan

## Abstract

Psoriasis, a skin inflammatory disease, originates from dysregulated interactions of the immune system. Cadmium, an environment pollutant, increases the levels of inflammation markers and influences the immune system. To clarify the relationship between cadmium and psoriasis, 5,927 participants, ≥20 years, in the National Health and Nutrition Examination Survey (NHANES) 2003–2006 were studied. Psoriasis severity was assessed using self-reported dermatology questionnaires. Cadmium was measured using blood chemistry. Three adjusted models were applied for the interaction between serum cadmium and severity of psoriasis. Psoriasis patients had significantly higher blood cadmium (0.67 vs. 0.52 μg/L, *p* = 0.006). There was a strong linear increase in predicted blood cadmium values with an increase in severity of psoriasis (*p* for trend = 0.002). The β coefficient of the predicted serum cadmium in the “few patches to extensive psoriasis” group was 0.234 (*p* = 0.002) after adjusting covariates. Participants with severe psoriasis have higher blood cadmium. Environmental exposure to cadmium can predispose to the worsening of psoriasis. Although there are still limitations in this study, such as not included treatment data, these results have substantial public health implications for the general population, as they demonstrate the importance of cadmium exposure prevention, particularly among psoriasis patients.

## Introduction

Psoriasis is a chronic inflammatory skin disorder that appears in the form of well-demarcated, scaly plaques. Psoriasis is common and affects approximately 2–3% of the white population^1^. Higher rates of cardiovascular morbidity and mortality have been reported in psoriasis patients because of systemic chronic inflammation^2–4^. Furthermore, metabolic syndrome, smoking, and obesity are known to be more prevalent in psoriasis patients^2, 5^. Dose-response relationships between higher prevalence of metabolic syndrome components and more severe psoriasis were established^5^. In addition to inflammation, psoriasis is also considered to originate from unbalanced the immune system with resident cells of the skin^6^. Cadmium is a ubiquitous environmental contaminant and is toxic even at low levels. Chronic exposure to cadmium involves a variety of pathological conditions and constitutes a serious environmental health problem. The usual sources of exposure in the general population are tobacco smoke and diet. Contaminated air and dust can be also important sources in communities near industrial sites^7^ and main-road sites^8^. Cadmium can increase the risks of multiple-organ disease and metabolic syndrome. Cadmium also causes the elevation of inflammation markers and influences the immune system^9^. The relationship between cadmium and psoriasis, an inflammatory skin disorder, is poorly understood. Accordingly, we hypothesized that cadmium would be positively associated with severity of psoriasis.

## Results

### Participants

The overall response numbers for the National Health and Nutrition Examination Survey (NHANES) 2003–2006 was *N* = 20,470. We excluded participants <20 and >60 years old (*n* = 10,450) and those whose self-reported dermatology questionnaires lack psoriasis status data (*n* = 3,488) or results of laboratory and clinical examinations (*n* = 605). This resulted in 5,927 eligible subjects (2,777 men and 3,150 women) with complete information.

### Characteristic of the study population

The characteristics of the participants stratified by psoriasis are summarized in Table 1. Among the 5,927 individuals were included in the analyses, of whom 150 reported having ever been diagnosed by a healthcare provider as having psoriasis, which the prevalence at baseline was 2.5%. Significant findings from analysis showed individuals with psoriasis to have a higher mean age, to more often be non-Hispanic white, be higher education level, be obese, be former smokers, have a greater smoking dose, have higher rate of cancer and arthritis. Participants with psoriasis had a borderline significantly higher rate of hypertension (10.9% vs. 16%, *p* = 0.065). Significantly higher blood cadmium was noted in the psoriasis group (0.67 vs. 0.52 μg/L, *p* = 0.006) and other metals were statistically significant. The white blood cell count and C-reactive protein measures of general health status were lower for those with psoriasis, although none were statistically significant.

**Table 1:** Characteristics of study participants.

Variables	Never diagnosed with psoriasis N = 5,777	Psoriasis N = 150	*p* value
Continuous variables			
Age (years), mean (SD)	37.63 (11.23)	40.37 (10.74)	0.003
Systolic blood pressure, mean (SD)	118.26 (15.66)	119.45 (16.08)	0.41
Diastolic blood pressure, mean (SD)	70.26 (12.42)	73.15 (12.02)	0.011
Waist circumference (cm), mean (SD)	96.85 (15.78)	101.92 (19.18)	<0.001
Body mass index (kg/m^2^), mean (SD)	28.67 (6.86)	30.68 (7.45)	<0.001
Serum glucose (mg/dL), mean (SD)	94.39 (30.17)	94.26 (25.21)	0.96
Serum total cholesterol (mg/dL), mean (SD)	201.55 (44.26)	204.73 (39.46)	0.39
Serum triglycerides (mg/dL), mean (SD)	146.33 (144.9)	158.5 (152.45)	0.31
Serum creatinine (mg/dL), mean (SD)	0.86 (0.3)	0.89 (0.19)	0.24
White blood cell count (1000 cells/uL), mean (SD)	7.52 (2.36)	7.79 (2.41)	0.16
C-reactive protein (mg/dL), mean (SD)	0.46 (0.85)	0.55 (1.03)	0.17
Serum total bilirubin (mg/dL), mean (SD)	0.72 (0.34)	0.73 (0.29)	0.76
Serum cadmium (ug/L), mean (SD)	0.52 (0.62)	0.67 (0.95)	0.006
Serum lead (ug/L), mean (SD)	1.69 (1.57)	1.6 (1.21)	0.495
Serum mercury, total (ug/L), mean (SD)	1.56 (2.19)	1.83 (2.34)	0.139
Pack-yrs smoking, mean (SD)	14.48 (20.33)	20.97 (50.09)	0.006
Categorical variables			
Sex			0.298
Men, *n* (%)	2713 (46.9)	64 (42.6)	
Women, *n* (%)	3064 (53.1)	86 (57.4)	
Race and Hispanic origin			<0.001
Non-Hispanic white, *n* (%)	2721 (47.1)	102 (68)	
Non-Hispanic black, *n* (%)	1319 (22.8)	27 (18)	
Mexican American, *n* (%)	1250 (21.6)	11 (7.3)	
Years of Education, y			0.017
<12, *n* (%)	1369 (23.7)	23 (15.3)	
>=12, *n* (%)	4405 (76.3)	127 (84.7)	
Annual Family Income, $			0.067
<35,000	2523 (43.6)	57 (38)	
>=35,000	3005 (52)	89 (59)	
Hypertension, *n* (%)	631 (10.9)	24 (16)	0.065
Coronary heart disease, *n* (%)	67 (1.1)	3 (2)	0.127
Cancer or malignancy, *n* (%)	204 (3.5)	11 (7.3)	0.044
Arthritis, *n* (%)	816 (14.1)	48 (32)	<0.001
Nonsmokers, *n* (%)	3170 (54.9)	72 (48)	0.095
Current smokers, *n* (%)	1561 (27)	40 (26.7)	0.923
Former smokers, *n* (%)	1047 (18.1)	38 (25.3)	0.024
Alcohol drinking, *n* (%)	3811 (65.9)	101 (67.3)	0.643

### Severity of psoriasis and blood cadmium

**Figure 1:**
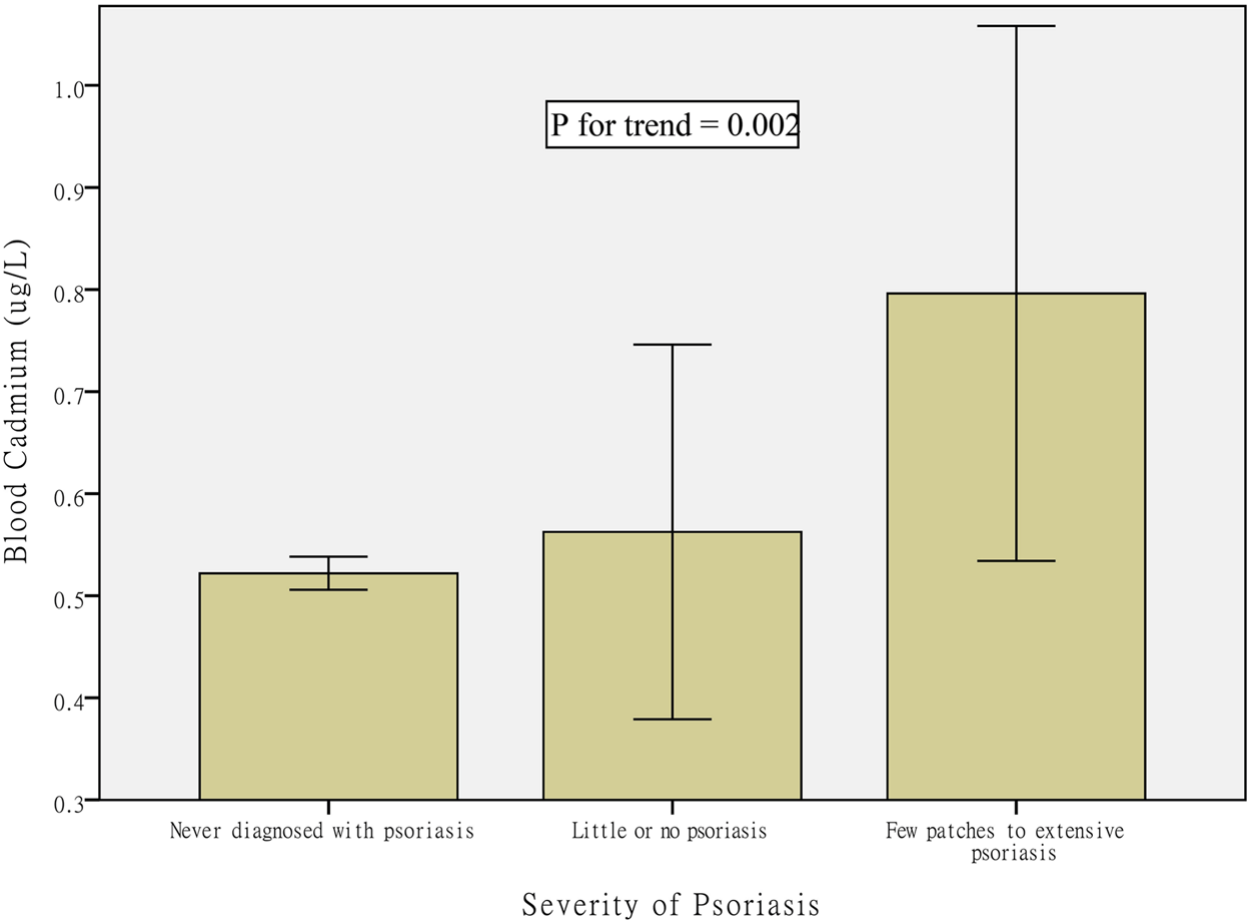
Psoriasis and Cadmium. Mean values of blood cadmium across severity of psoriasis.

Results from the models examining the association between severity of psoriasis and predicted blood cadmium values are presented in Table 2. Blood cadmium level was positively associated with “presence of psoriasis” after adjusting for covariates. There was a strong linear increase in predicted blood cadmium values with increased severity of psoriasis (Fig. 1, *p* for trend = 0.002). The β coefficient of the predicted serum cadmium in the “few patches to extensive psoriasis (BSA ≥ 1%)” group was 0.261 (*p* = 0.001) after adjusting for age, gender, race/ethnicity, family income, education level (model 1). Moreover, after additionally adjusting for other covariates in models 2 and 3, the coefficient showed little difference, and the positive correlation remained (all *p* ≤ 0.005). These findings indicated that severity of psoriasis positively correlated with blood cadmium.

**Table 2:** Regression coefficients of presence and degree of psoriasis with blood cadmium.

	Presence of Psoriasis (*n* = 150)		Degree of psoriasis					*p* for trend
			Never diagnosed with psoriasis (*n* = 5,777)	No or little psoriasis (<1% BSA) (*n* = 83)		Few patches to extensive psoriasis (≥1% BSA) (*n* = 67)		
	β (95% CI)	*p-*values	β (95% CI)	β (95% CI)	*p-*values	β (95% CI)	*p-*values	
Unadjusted	0.164 (0.054, 0.275)	0.004	**Reference**	0.064 (−0.085, 0.213)	0.403	0.284 (0.122, 0.446)	0.001	0.001
Model 1	0.145 (0.037, 0.253)	0.008	**Reference**	0.048 (−0.098, 0.193)	0.521	0.261 (0.103, 0.419)	0.001	0.002
Model 2	0.154 (0.05, 0.258)	0.004	**Reference**	0.058 (−0.082, 0.197)	0.418	0.26 (0.11, 0.413)	0.001	0.001
Model 3	0.151 (0.049, 0.252)	0.004	**Reference**	0.073 (−0.063, 0.209)	0.294	0.234 (0.085, 0.382)	0.002	0.001

Adjusted covariates: Model 1 = age, gender, race/ethnicity, family income, education level. Model 2 = Model 1 + (hypertension, coronary heart disease, arthritis, cancer) + (Body mass index, waist circumference, alcohol consumption, former smoker, smoking dose). Model 3 = Model 2 + (white blood cell count, C-reactive protein, blood lead, blood mercury).

CI, confidence interval.

### Blood cadmium and psoriasis

The association between the blood cadmium levels and predicted psoriasis ever in life is presented in Table 3. In the multiple logistic analysis, participant with higher blood cadmium have higher predicted psoriasis (all *p* < 0.05). In the unadjusted analysis, the OR of the predicted psoriasis with blood cadmium were 1.336 (*p* = 0.004). After additional adjustment, the OR of the predicted psoriasis with blood cadmium was 1.341, respectively (*p* = 0.009). Collectively, these results showed that blood cadmium have higher predicted psoriasis.

**Table 3:** Association between blood cadmium level and psoriasis.

Models	OR (95% CI)	*p*-value
Unadjusted	1.336 (1.1, 1.63)	0.004
Model 1	1.312 (1.07, 1.61)	0.01
Model 2	1.317 (1.60, 1.64)	0.013
Model 3	1.341 (1.08, 1.67)	0.009

Adjusted covariates: Model 1 = age, gender, race/ethnicity, family income, education level. Model 2 = Model 1 + (hypertension, coronary heart disease, arthritis, cancer) + (Body mass index, waist circumference, alcohol consumption, former smoker, smoking dose). Model 3 = Model 2 + (white blood cell count, C-reactive protein, blood lead, blood mercury).

OR, odds ratio; CI, confidence interval.

## Discussion

This study demonstrated a positive association between blood cadmium and psoriasis in a U.S. population. After controlling for multiple confounding factors, the positive correlation remained. Participants with psoriasis tended to have higher blood cadmium, especially in severe psoriasis. Higher blood cadmium also can predict higher rate of psoriasis. Most prior studies generally focused on occupational bio-monitoring and non-occupationally exposed populations were small. Therefore, previous studies were limited ability to examine a wide range of exposure strength and decency.

Psoriasis, a chronic, immune-mediated inflammatory skin disease, affects 2–3% of the general population^10^ and is a significant public health problem^11^. The typical type of psoriasis manifests as red, well-demarcated, scaly plaques about the size of a palm. Psoriasis is a multifactorial disease with extrinsic and intrinsic factors. It presents with simultaneous appearance of inflammation, epidermal hyperproliferation, and angioneogenesis^6^. In the past, psoriasis was considered as a disease of hyperproliferation, but recently, it is regarded as an immune-mediated disease^12^. Imbalance in the Th1/Th2 immune system with Th1 predominance plays a central pathogenetic role in psoriasis. Psoriasis is a chronic inflammatory skin disease mediated by Th1 and Th17, which have critical roles in thrombosis and atherosclerosis^13^.

Recently, psoriasis has been considered a systemic disease rather than a single-organ disease. Patients diagnosed with psoriasis are known to be at an increased risk of metabolic syndrome and cardiovascular disease^14, 15^. Previous epidemiological studies have demonstrated higher cardiovascular risk factors in psoriasis patients, including metabolic syndrome, diabetes mellitus, hypertension, cigarette smoking, obesity, insulin resistance, and dyslipidemia^16^. Lai and Yew^17^ evaluated a national database and found that psoriasis is an independent risk factor for cardiovascular disease. Armstrong *et al*.^18^ reviewed and found that psoriasis patients have higher prevalence of metabolic syndrome. Patients with more severe psoriasis also have higher odds of metabolic syndrome than those with milder psoriasis. The possible pathophysiology might be overlapping inflammatory pathways and genetic predisposition^19^. The potential biological mechanisms that link psoriasis and metabolic syndrome are chronic Th1- and Th17-mediated inflammation with dysregulation of cytokines, including tumor necrosis factor α (TNF-α) and interleukin 6 (IL-6). Such biological mechanisms not only promote epidermal hyperplasia in psoriasis, but may also antagonize insulin signaling, alter adipokine expression, and increase risk of insulin resistance and obesity^20^. A recent research presented that higher white blood cell (WBC), C- reactive protein (CRP) and lower serum total bilirubin are associated with the enhancement of the inflammatory response in psoriasis^21^. In our study, the white blood cell count and C-reactive protein measures of general health status were lower for those with psoriasis, although no statistically significant.

Cadmium, an environment pollutant, has a biological half-life of more than 10 years in the whole body. People are exposed to cadmium mainly from respiratory and digestive tracts. Occupational exposure is the most common cause of elevated cadmium levels. Cadmium is refined and used in pigments, batteries, plating and coatings, plastic stabilizers, photovoltaic devices, nonferrous alloys, and others^7^. Non-occupational and environmental exposures, such as various foods, contaminated water, smoke, and contaminated dust, also increase body cadmium levels. Different from other metals, cadmium compounds are highly soluble and high soil-to-plant transfer rate^22^. Cadmium compounds can be taken up by plants resulting in storage in crops for food and feed production. Cereals and vegetables are the main source of dietary cadmium in humans^23^. Smoking is also a main mechanism of cadmium intake by humans^24^, and it is more serious than the presence of cadmium in food. In indoor environments, passive exposure to cadmium is from sidestream smoke^25^. In our study, the prevalence of former smoker in psoriasis group is higher than non-psoriasis group and the accumulated smoking dose is also obviously higher. It may be one plausible reason to explain the association between psoriasis and cadmium.

Cadmium accumulates in the body with age, and only a small percentage (0.01–0.02%) of the body burden could be excreted per day^26^. Cadmium can cause early kidney damage^27^. The International Agency for Research on Cancer classified cadmium and its compounds as group 1 carcinogens to humans. Navas-Acien *et al*.^28^, by analyzing the NHANES 1999–2006 data set, reported that a high level of blood cadmium can be a chronic kidney disease risk factor in the general population. Besides nephrotoxicity, growing studies suggest that chronic exposure to cadmium may increase risk of prediabetes^26^, diabetes^29^ and metabolic syndrome^30^. Higher concentrations of cadmium were reported in the blood, urine, and scalp hair of patients with diabetes, compared with an age-matched control group^31^. Cadmium can disturb glucose homeostasis, reduce liver glycogen, enhance gluconeogenic potential of hepatic tissue, and inhibit insulin release, which results in hyperglycemia in chronically exposed neonatal rats^32^. Cadmium-induced renal tubular dysfunction was potentiated by diabetes in groups of the general population^31^.

Cadmium is also harmful to the skin. After daily administration of cadmium chloride solution to the shaved skin of mice and rats for 10 days, the skin showed acanthosis and hyperkeratosis with occasional ulcerative changes. Percutaneous absorption was proven by the increased cadmium concentration in the blood, liver, and kidney^33^. Lesions on the skin and tumors in the scrotum in the rats were observed after dermal application of cadmium.

Cadmium can accumulate intracellularly due to its binding to cytoplasmic and nuclear material. When elevated cytotoxic concentrations, cadmium inhibits the biosyntheses of DNA, RNA, and protein. Cadmium also causes DNA strand breaks, lipid peroxidation, and chromosome aberrations^34^. Otherwise, cadmium plays an important role for oxidative stress and induces toxicity and pathogenesis^35^ at cellular level and cause multiple diseases. The production of reactive oxygen species (ROS) induced by cadmium has toxic effects in many tissues and organs^36^.

The skin, continuously exposed many environmental stresses generating ROS, is a potential target for oxidative injury^37^. ROS mediate oxidative damage and relates lipid peroxidation, DNA modification, and secretion of inflammatory cytokines^38^. Dipali PK and colleagues provided an evidence for increased ROS production and decreased antioxidant defenses in psoriasis^39^. Cadmium seems to be linked with the enhancement of ROS and decreased antioxidant potential in psoriasis.

Moreover, zinc, an important component of healthy skin, may be deficient due to slowly developing cadmium toxicity. Cadmium can interfere with zinc absorption, distribution and inhibit zinc activities^40^. Michaëlsson *et al*. reported epidermis, papillary dermis and serum zinc concentration was decreased in patient with psoriasis^41^. Zinc supplement, oral^42^ or topical use^43^ had been proved to improve the condition of psoriasis.

Long-term exposure to cadmium may be related to changes in immune response^44^. Tsangaris and Tzortzatou-Stathopoulou^45^ noted that a differential Cd^2+^-induced apoptotic effect may disturb immune system development and normal growth. Olszowski *et al*.^9^, in their review, concluded that cadmium causes the upregulation of many markers of inflammation, such as IL-6, TNF-α, IL-1β, IL-8, and CRP; thus, cadmium appears to possess pro-inflammatory properties.

Emerging evidence indicate that accumulation cadmium may affect psoriasis through mechanisms, such as induce oxidative stress, cadmium-induced zinc deficiency, changes in immune response, and upregulation inflammation markers.

Psoriasis and a high level of blood cadmium can cause skin keratosis and are related with metabolic syndrome and prediabetes. Severity of psoriasis was strongly related with higher blood cadmium concentration in the general population. A 3-year follow-up study evaluated participants with psoriasis from the Pakistan Cement Factory area. The mean values of cadmium were significantly higher in the scalp, hair, blood, and urine samples of patients with mild and severe psoriasis, as compared with control^46^. However, the Pakistan study has no possible mechanism of interaction and participants were limited to those in the Pakistan Cement Factory area.

In addition, cigarette smoking is considered a risk factor for both development and exacerbation of psoriasis. Fortes *et al*.^47^ investigated 818 Italian participants with psoriasis and found that smoking is associated with the clinical severity of psoriasis. Many metals were found in cigarette smoke, and cadmium is the best-studied metal from cigarette smoke^31^. In our study, smoking is also more prevalent in psoriasis group. The association between blood cadmium level and psoriasis severity remained significant even after additional adjustment for smoking status and smoking dose.

In the present study, we analyzed a U.S. general population, and our results contributed to the literature examining the association between psoriasis and cadmium. Severe psoriasis was strongly related with higher blood cadmium in the general population. Higher blood cadmium is associated with predicting psoriasis. The association remained despite adjusting for multiple confounders.

There were some limitations of the present study. First, this was a cross-sectional study and did not demonstrate a cause-and-effect relationship. The true causality and possible mechanisms underlying the relationship between blood cadmium and psoriasis should be further examined. Second, self-reported psoriasis was used instead of structured diagnostic scales, such as the Psoriasis Area Severity Index, might affect the validity of the findings. However, in such large population-based surveys, measure of self-reported psoriasis has been extensively utilized^48, 49^, valid and reliable^50^. Nevertheless, utilization of more comprehensive scales or indices should be considered in future studies. The Third, this study did not evaluate urinary cadmium owing to limited available data. Additionally, the correlation of cadmium with air pollution, occupation, secondhand smoke and dietary data are not included in this study. Since the investigating the link between blood cadmium levels and psoriasis development is significant, residing close to a main road, dietary, occupation, secondhand smoke exposure and treatment for psoriasis should be taken into account as a co-founding factor. Fourth, only a limited number of inflammatory markers were adjusted for in this study. A larger number of inflammatory markers, such as TNF-α and IL-6, should be included and controlled to reduce selection bias. Finally, despite the large population sample, the number of psoriasis patients was low. In our study, the prevalence of psoriasis is 2.5% and is significantly more prevalent among non-Hispanic whites, which is compatible with previous studies^51^. More longitudinal studies with a more subjects and different populations are necessary to confirm and extend the present findings.

## Conclusions

We demonstrated that psoriasis was independently associated with high blood cadmium. In a U.S. general population, participants with severe psoriasis have higher blood cadmium. Environmental exposure to cadmium may compromise immunity, and microenvironmental perturbation can predispose to the worsening of psoriasis. Given the widespread exposure to cadmium and the increasing cases of psoriasis, which increase the worldwide burden of metabolic syndrome, these data have substantial public health implications for the general population. Smoking, sidestream smoke and exposure to cadmium can exacerbate metabolic syndrome, diabetes, and cardiovascular disease, and the results of this study demonstrate the importance of smoking cessation, avoidance of sidestream smoke, and prevention of exposure to cadmium, particularly among psoriasis patients. Further studies are needed to confirm this association and to investigate the mechanisms involved in the effects of cadmium on psoriasis.

## Methods

### Study population

We used data from a cross-sectional analysis of the National Health and Nutrition Examination Survey (NHANES). NHANES, a program under the Centers for Disease Control and Prevention (CDC), is executed every 2 years and uses a multistage, stratified design to assess the health and nutritional status of adults and children in the United States. NHANES data have been released every 2 years since 1999. NHANES conducts sampling to represent appropriate samples of the U.S. population. The Institutional Review Board of NCHS approved the NHANES. A description of the laboratory analysis can be found on the NHANES resources. We combined NHANES data sets from 2003 to 2004 and from 2005 to 2006 to increase statistical reliability based on the NHANES analytic guidelines and the degree of psoriasis is only available during 2003–2006. Participants aged 20–59 years in the 2003–2004 and 2005–2006 NHANES cycles were examined. The institutional review board of the National Center for Health Statistics approved the NHANES 2003–2006 study, and ﻿all participants provided informed written consent before the initiation of the study.

### Measures

In the 2003–2006 survey, information regarding self-reported dermatology questionnaires included the participants’ psoriasis status. To assess psoriasis diagnosis and extent of psoriatic skin lesions, the participants were also asked the following questions: “Have you ever been told by a health care provider that you had psoriasis?” and for those who responded “yes,” the following question was asked: “Do you current have (i) little or no psoriasis (<1 hand palm); (ii) only a few patches (that could be covered between 1–2 hand palms); (iii) scattered patches (that could be covered between 3–10 hand palms); and (iv) widespread psoriasis (covering large areas of the body, that would be more than 10 hand palms)?” The type of psoriasis was not assessed in the NHANES data. Based on previous studies^37^, we combined (ii), (iii) and (iv) due to small frequency counts.

Blood cadmium, lead and total mercury concentrations were examined at the Environmental Health Sciences Laboratory of the CDC National Center for Environmental Health after confirming the use of extensive quality control procedures and no background contamination. A PerkinElmer Model SIMAA 6000 simultaneous multielement atomic absorption spectrometer (PerkinElmer, Waltham, MA, USA) and inductively coupled plasma-mass spectrometer was used for blood cadmium, lead and total mercury measurements. The detection limit for metals was constant in the data set. In cases where the result was below the limit of detection, the value for that variable is the detection limit divided by the square root of 2. Detailed instructions on specimen collection and processing can be found on the NHANES website.

Chemical analysis of total cholesterol and triglycerides was conducted enzymatically with a Hitachi-704 Analyzer (Roche Modular P chemistry analyzer, Indianapolis, IN, USA) at the Lipoprotein Analytical Laboratory of the University of Minnesota. Serum C-reactive protein (CRP) levels were determined using latex-enhanced nephelometry. Complete blood count measurements with five-part differential in whole blood were determined according to the Beckman Coulter method of counting and sizing, in combination with an automatic diluting and mixing device for sample processing. Other routine biochemistry profiles were analyzed using Beckman Synchron LX20 and Beckman UniCel® DxC800 Synchron (Beckman Coulter, Fullerton, CA, USA).

The participants’ information such as age, sex, race/ethnicity, education level, annular family income and body measurements (including height, weight, and waist circumference), blood pressure, and medical conditions were collected in mobile examination centers. Body mass index (BMI) was calculated by dividing the participant’s weight in kilograms by the square of their height in meters (kg/m^2^). The presence of hypertension was based on a self-reported doctor’s diagnosis, use of antihypertensive medications, or an average blood pressure ≥140/90 mmHg. Trained NHANES staff measured waist circumference using standard protocols. Smoking status was based on question “Lifetime use of ≥100 cigarettes” and “Do you now smoke?”. Ever-smokers indicating smoking cigarettes “every day” or “some days” were categorized as current smokers. Former smokers were ever-smokers who self-reported currently smoking “not at all”. Years of smoking were ascertained for ever-smokers from age of initiation and age at interview or age of cessation, for current and former smokers respectively. Smoking rate was obtained by NHANES as cigarettes per day and converted to packs by assuming 20 cigarettes per pack. Smoking dose was quantified in pack-years (py) with 1 py which defined as smoking one pack per day for one year. Alcohol use was defined as having at least 12 drinks in the past year. Medical histories of coronary artery disease, cancer or malignancies and arthritis were self-reported. Detailed specimen collections and processing instructions are provided in the NHANES Laboratory Procedures Manual, which is available on the NHANES website.

### Statistical analysis

The NHANES data sets from 2003 to 2004 and from 2005 to 2006 were combined according to the NHANES analytic guidelines. The predicted values of blood cadmium were divided into three groups: “never diagnosed with psoriasis,” “little or no psoriasis,” and “few patches to extensive psoriasis.” Student *t*-test was used to analyze continuous data, and χ^2^ test was used to analyze categorical data.

The associations between blood cadmium and severity of psoriasis were determined by multiple linear regression. The *p*-values for the trend tests were determined by treating the severity of psoriasis as a continuous variable (1–3) to observe the associations between increased severity of psoriasis and blood cadmium. Logistic regression models were constructed to obtain both unadjusted and adjusted odds ratio (OR) and 95% confidence interval (CI) for the odds of having psoriasis. Based on previous studies^11, 15, 21, 30^, influential demographic factors and clinical standpoints can influence the results and were thus used in covariate adjustment. Four regression models were constructed, and of these, one was unadjusted and the others were adjusted: model 1 was included age, gender, race/ethnicity, family income and education level adjustment; model 2 included model 1 + hypertension, coronary heart disease, arthritis, cancer, body mass index, waist circumference, alcohol consumption, former smoker and smoking dose adjustment; model 3 included model 2 + white blood cell count, C-reactive protein, blood lead and blood mercury adjustment. All statistical analyses were performed using SPSS (version 18.0 for Windows; IBM, Armonk, NY, USA). Two-sided *p*-values < 0.05 indicate significant differences.

### Ethics statement

The National Center for Health Statistics Institutional Review Board approved the 2003–2006 NHANES study, and written informed consent was obtained from all participants before the study. However, this study was exempt from IRB review because we analyzed an openly unidentifiable, available online database. All methods in this study were performed based on the relevant guidelines and regulations.
